# Clinical Significance of Plasma Apolipoprotein-AII Isoforms as a Marker of Pancreatic Exocrine Disorder for Patients with Pancreatic Adenocarcinoma Undergoing Chemoradiotherapy, Paying Attention to Pancreatic Morphological Changes

**DOI:** 10.1155/2019/5738614

**Published:** 2019-04-04

**Authors:** Aoi Hayasaki, Yasuhiro Murata, Masanobu Usui, Taemi Hibi, Takehiro Fujii, Yusuke Iizawa, Hiroyuki Kato, Akihiro Tanemura, Yoshinori Azumi, Naohisa Kuriyama, Masashi Kishiwada, Shugo Mizuno, Hiroyuki Sakurai, Katsunori Uchida, Shuji Isaji

**Affiliations:** ^1^Department of Hepatobiliary Pancreatic and Transplant Surgery, Mie University, Hepatobiliary Pancreatic and Transplant Surgery, Mie University School of Medicine, 2-174 Edobashi, Tsu, Mie 514-8507, Japan; ^2^Department of Oncologic Pathology, Mie University, Hepatobiliary Pancreatic and Transplant Surgery, Mie University School of Medicine, 2-174 Edobashi, Tsu, Mie 514-8507, Japan

## Abstract

**Background:**

Circulating apolipoprotein-AII (apoAII-) ATQ/AT is a potential useful biomarker for early stage pancreatic ductal adenocarcinoma (PDAC), but its clinical significance in PDAC patients remains uncertain. The aim of the current study was to assess the usefulness of apoAII-ATQ/AT as a surrogate for the effect of chemoradiotherapy (CRT) and its association with pancreatic exocrine disorder, paying attention to morphological changes of the pancreas.

**Methods:**

In the 264 PDAC patients who were enrolled in our CRT protocol, the following parameters were measured at specified time points before and after CRT: serum levels of albumin, total cholesterol, and amylase as indices of pancreatic exocrine function, serum levels of CA19-9, and the pancreatic morphology including tumor size (TS), main pancreatic duct diameter (MPDD), and pancreatic parenchymal volume excluding tumor volume (PPV) by using computed tomography (CT) images. Plasma apoAII-ATQ/AT levels were simultaneously measured with enzyme-linked immunosorbent assay in 4 healthy volunteers and the 44 PDAC patients before and after CRT. Plasma apoAII-ATQ/AT levels after CRT were analyzed according to small/large-MPDD and small/large-PPV groups based on their median values after CRT. Plasma samples after CRT were measured after incubation with human pancreatic juice (PJ) to examine the relevance between apoAII isoforms and circulating pancreatic enzymes.

**Results:**

The serum levels of albumin, amylase, CA19-9, TS, MPDD, and PPV after CRT were significantly lower than those before CRT (median, before vs. after: 3.9 g/dl, 74 U/l, 180.2 U/ml, 58.1 mm, 4.0 mm, and 34.8 ml vs. 3.8, 59, 43.5, 55.6, 3.6, and 25.2). ApoAII-ATQ/AT levels (median, *μ*g/ml) of PDAC patients before CRT were significantly lower than those in healthy volunteers: 32.9 vs. 61.2, and unexpectedly those after CRT significantly decreased: 14.7. The reduction rate of apoAII-ATQ/AT was not correlated with those of CA19-9 and TS, indicating that apoAII-ATQ/AT is not a tumor-specific marker. On the other hand, the patient group with large MPDD and small PV exhibited higher apoAII-ATQ levels than those with small MPDD and large PPV. The incubation of plasma samples after CRT with PJ did not alter apoAII-ATQ/AT and apoAII-AT levels but significantly decreased apoAII-ATQ levels, suggesting that circulating pancreatic enzymes markedly influenced apoAII-ATQ levels.

**Conclusions:**

ApoAII-ATQ/AT levels are not useful for evaluation of clinical effect of CRT for PDAC, but apoAII isoforms are very useful to assess pancreatic exocrine disorder because pancreatic atrophy and insufficient secretion of circulating pancreatic enzymes are considered likely to influence apoAII-ATQ levels.

## 1. Introduction

Pancreatic ductal adenocarcinoma (PDAC) is one of the most lethal malignant tumors. Of those afflicted, 10-20% are eligible for potentially curative resection, and up to 80% of patients present with locally advanced or metastatic disease at diagnosis [1]. To improve PDAC patients' prognosis, early detection and effective multidisciplinary treatment are necessary. Circulating apolipoprotein-AII (apoAII-) ATQ/AT is one of the apoAII isoforms and a potential biomarker for screening patients for early stage PDAC. It was previously reported that the levels of apoAII-ATQ/AT in plasma samples from PDAC patients were significantly lower than those from healthy controls [2]. ApoAII is a protein consisting of 77 amino acids mainly produced in the liver and forms a homo/heterodimer via an N-terminal disulfide bond. There are five circulating apoAII isoforms: apoAII-ATQ/ATQ, -ATQ/AT, -AT/AT, -AT/A, and -A/A, which are characterized by the truncation of varying numbers of amino acids from the C-terminus of the apoAII homodimer. ApoAII-ATQ and -AT can be measured by sandwich enzyme-linked immunosorbent assays (ELISAs) that were developed and reported by Honda et al. in 2015, and apoAII-ATQ/AT was calculated by using this formula: apoAII-ATQ/AT (*μ*g/ml) = ELISA ApoAII√(ATQ*∗*AT) [3]. Initial cleavage of a C-terminal glutamine (Q) in apoAII-ATQ/ATQ by carboxypeptidases might prime apoAII protein to further proteolytic digestion, and this process forms five circulating apoAII isoforms in the above order [4].

Among carboxypeptidases, carboxypeptidase A is a digestive enzyme that is primarily synthesized mainly by the pancreas as a zymogen precursor (procarboxypeptidase A), which is converted into the active form of carboxypeptidase A (active-carboxypeptidase A) by the reaction of trypsin in the intestinal lumen. Total carboxypeptidase A (pro- and active-carboxypeptidase A) is increased both in patients with PDAC and in those with acute pancreatitis, while high levels of active-carboxypeptidase A is produced in patients with acute pancreatitis. Therefore, the active-/total-carboxypeptidase A ratio was significantly lower in the patients with PDAC compared with those with acute pancreatitis [5, 6]. These facts let us speculate that the status of pancreatic enzyme secretion such as carboxypeptidase A into the blood stream influences the distribution of apoAII isoforms in plasma for PDAC patients and the levels of apoAII-ATQ/AT. However, its clinical usefulness and underlying significance other than early diagnosis of PDAC have not been elucidated.

Most patients with initially locally advanced PDAC receive nonoperative treatment, such as chemotherapy and chemoradiotherapy (CRT). Treatment strategy of chemotherapy and/or CRT followed by surgery allows for the identification of patients with occult metastatic disease, and R0 resection rate may be increased in the patients with locally advanced PDAC who show favorable clinical response to nonoperative treatment and subsequently undergo curative-intent surgery, resulting in a reduced risk for local recurrence and improvement of prognosis [7–9]. However, it remains difficult to select the patients who are suitable for curative-intent surgery among those who have received nonoperative treatment because of lack of useful surrogate markers for treatment effects.

To the best of our knowledge, there have been no studies on the clinical significance of apoAII-ATQ/AT as a biomarker to evaluate the treatment effects of CRT in patients with PDAC, although it has been reported that the levels of apoAII-ATQ/AT from patients with PDAC are significantly lower than those from healthy controls. We therefore first anticipated that plasma apoAII-ATQ/AT might be significantly increased according to the degree of treatment response, if it serves as a tumor-specific marker. The aim of the current study was to assess clinical utility of apoAII-ATQ/AT as a surrogate for CRT effects and its association with pancreatic morphological changes reflecting pancreatic exocrine functional impairment during CRT in patients with PDAC including locally advanced tumor.

## 2. Patients and Methods

### 2.1. Study I: Serum Marker of Pancreatic Exocrine Function and Morphological Features before and after CRT

The subjects of the current study were 264 patients with cytology or histology proven PDAC who were prospectively enrolled into the CRT protocol at Mie University Hospital [10] from February 2005 to May 2016. There were 165 male and 99 female patients with ages ranging from 41 to 87 years (median age: 68.0 years). Computed tomography (CT) was performed just before the initiation of CRT (before CRT) and at the time of reevaluation (after CRT) according to a defined pancreas protocol as four-phase dynamic multidetector 64-row contrast-enhanced CT (MD-CT) with thin slices every 1.00 or 1.25 mm. The tumor location and initial resectability were determined according to the seventh edition of General Rules for the Study of Pancreatic Cancer published by the Japan Pancreas Society in 2016 [11]. They consisted of 174 pancreatic head, 46 pancreatic body, and 44 pancreatic tail cancers. In terms of the initial resectability classification, there were resectable (R) PDAC in 52 patients and borderline resectable (BR) in 74 including portal vein invasion alone (BR-PV) in 28 and arterial invasion (BR-A) in 46, locally advanced unresectable (UR-LA) in 117, and unresectable with distant metastasis (UR-M) in 21. As for the CRT regimen, 97 patients received gemcitabine-based CRT protocol, and 167 received S-1 plus gemcitabine-based CRT protocol.

To assess the alteration of pancreatic exocrine function after CRT, serum levels of albumin, total cholesterol, and amylase were measured before and after CRT, because they were used as markers of the pancreatic exocrine function in some previous reports [12–14], although accurate methods for evaluation of pancreatic exocrine functions are not widely available in clinical practice. To assess the clinical effect of CRT, serum CA19-9 level and tumor size (TS: sum of short- and long-axis tumor diameter) on CT were measured at the time before CRT (pre-CA19-9 and pre-TS) and after CRT (post-CA19-9 and post-TS). Reduction rate in serum CA19-9 level [15] and TS were calculated as follows: (pre-CA19-9 - post-CA19-9) x100/ (pre-CA19-9) and (pre-TS - post-TS) x100/ (pre-TS) (%). To assess the alteration of morphological changes after CRT, main pancreatic duct diameter (maximum size) (MPDD) and pancreatic parenchymal volume excluding tumor volume (PPV) were measured on CT cross-section and by CT volumetry at the same time points before and after CRT, because it was considered that the alterations of MPDD and PPV were associated with the changes in pancreatic exocrine function [12, 16, 17] and that large MPDD and small PPV were most likely to reflect the impairment of pancreatic exocrine function in the patients with PDAC who were treated with CRT.

### 2.2. Study II: Alteration of Plasma ApoAII Isoforms before and after CRT

Among the 264 patients, measurements of plasma apoAII-ATQ and apoAII-AT (*μ*g/ml) with ELISA before and after CRT were performed in the 44 consecutive patients from February 2015 to May 2016. The plasma samples were collected and stored at −30°C until measurement. The plasma samples were diluted to 1/5000 before measurement. The concentration of apoAII-ATQ/AT was calculated by using this formula: apoAII-ATQ/AT (*μ*g/ml) = ELISA ApoAII√(ATQ*∗*AT) [3]. For the assessment of nutritional status, recently, CONUT (controlling nutritional status) score [18] and PNI (Prognostic Nutritional Index) [19] have been widely employed as simple methods and reliable indices. CONUT score consists of serum albumin, total lymphocyte, and total cholesterol, and PNI consists of serum albumin and total lymphocyte. Therefore, we examined the additional data on total lymphocyte and PNI in these 44 patients.

Among these 44 patients (R/BR-PV/BR-A/UR-LA/UR-M: 2/5/10/18/9), 13 (29.5%) (R/BR-PV/BR-A/UR-LA/UR-M: 0/5/4/2/2) underwent curative-intent pancreatectomy. In these 13 resected patients, the resected specimens were fixed in a formalin solution, sliced into 5 mm sections, and embedded in paraffin blocks. A 3 *μ*m section was obtained from each block and stained with hematoxylin and eosin. The pathological findings have been reviewed by the pathologist (K.U.) to assess the histological response and pancreatic parenchymal change in noncancerous area. The histological response was divided into two groups: high responders in which tumor destruction was more than 50%, and low responders in which tumor destruction was 50% or less [20].

To assess the relationship between alteration of apoAII-ATQ/AT and clinical effect of CRT, correlations between reduction rate in apoAII-ATQ/AT, which was calculated by the formula: (pre-apoAII-ATQ/AT - post-apoAII-ATQ/AT) x100/ (pre-apoAII-ATQ/AT) (%), and in serum CA19-9 level and TS were analyzed. Moreover, the 44 patients were divided into small/large-MPDD and small/large-PPV groups based on each median level after CRT (median, 3.65 mm and 29.5 ml, respectively). Plasma levels of apoAII-ATQ/AT, -ATQ, -AT were compared between small and large MPDD, or small and large PPV.

To clarify the relationship between the distribution of apoAII isoforms and circulating pancreatic enzymes which reflected pancreatic exocrine function, the plasma samples after CRT were diluted to 1/5000 and incubated with the same amount of pancreatic juice (PJ) diluted to 1/5000 at 37°C for 30 minutes, and their apoAII-ATQ/AT concentrations were subsequently measured. The PJ was obtained from the one patient who underwent subtotal stomach preserving pancreaticoduodenectomy for branch-duct intraductal papillary mucinous neoplasm (IPMN). The PJ was collected from an intraoperatively placed pancreatic duct stent and stored at −30°C until measurement. The value of apoAII-ATQ/AT was 1.5*μ*g/ml, suggesting that the pancreatic juice which was collected from the non-PDAC patients hardly contained apoAII isoforms.

**Figure 1:**
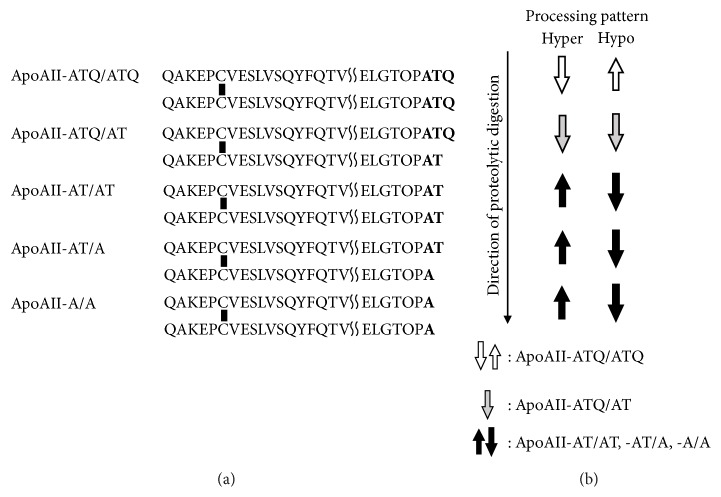
(a) Amino acid sequences of the five apoAII isoforms (ApoAII-ATQ/ATQ, -ATQ/AT, -AT/AT, -AT/A, and -A/A). Two peptides were dimerized via an N-terminal disulfide bond. (b) Direction of proteolytic digestion (long arrow) and the alteration of the isoforms (box arrows) in hyper- and hypoprocessing patterns (originally produced according to [4]).

To comprehend the alteration and distribution of five apoAII isoforms (Figure 1(a)), plasma apoAII-ATQ level was divided into the following two groups based on the median apoAII-ATQ level (36.3 *μ*g/ml) of healthy controls because apoAII-ATQ/ATQ was considered to sensitively reflect the two distinct processing patterns of apoAII isoforms as shown in Figure 1(b): (1) the group with hyperprocessing patterns in which the apoAII-ATQ level was very low (less than 36.3 *μ*g/ml) reflecting large amount of consumption of apoAII-ATQ/ATQ and (2) the group with hypoprocessing patterns in which the apoAII-ATQ level was very high (36.3 *μ*g/ml or more) reflecting little consumption of apoAII-ATQ/ATQ.

Informed consent was obtained from all patients, and this study was approved by the Ethics Committee of Mie University Graduate School of Medicine (No. 2955, 3045). The clinical and follow-up information was extracted from a prospectively maintained database at the department of Hepatobiliary Pancreatic and Transplant Surgery, Mie University, and verified by reviewing patient electric medical records.

Continuous variables were expressed as median (range) and were compared using the Mann-Whitney test and Wilcoxon signed rank test for the unpaired and paired comparisons of clustered data, respectively. Continuous variables more than three were compared by Kruskal-Wallis test. Correlation between the two variables was evaluated by Spearman rank correlation coefficient. All statistical analyses were performed with JMP® Pro 9.0.2 (SAS Institute Inc., Cary, NC). P values less than 0.05 were considered statistically significant.

## 3. Results

### 3.1. Study I: Serum Marker of Pancreatic Exocrine Function and Morphological Features before and after CRT

**Figure 2:**
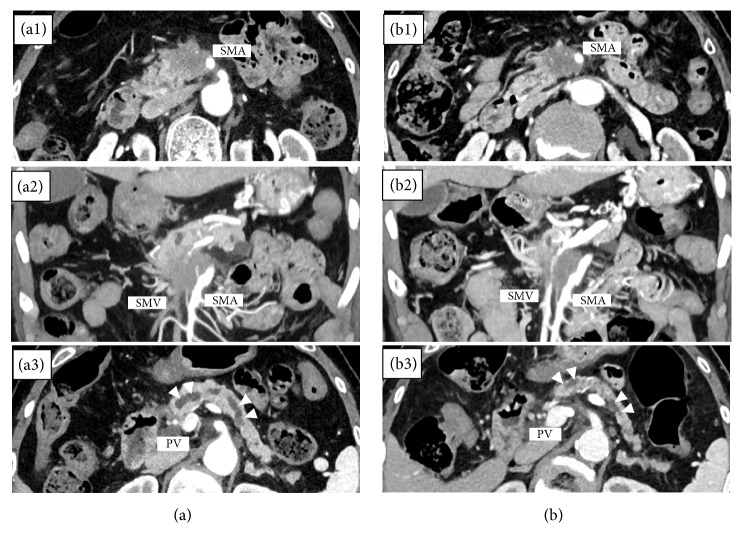
A case of UR-LA PDAC showing drastic reduction of TS, MPDD, and PPV after CRT ((a) before CRT and (b) after CRT. The time interval of CT images between (a) and (b) is 3 months). Arrow head shows main pancreatic duct (a1) and (b1). Cross-section CT images showing the reduction of TS (59.4→52.2 mm) after CRT (a2) and (b2). Coronal section CT images showing the reduction of portal vein invasion after CRT (a3) and (b3). Cross-section CT images showing the reduction of MPDD (7.6→3.7 mm) and PPV (39.8→29.3 ml) after CRT. UR-LA PDAC: unresectable pancreatic ductal adenocarcinoma, TS: tumor size (sum of short and long axis), MPDD: main pancreatic duct diameter, PPV: pancreatic parenchymal volume excluding tumor volume, and CRT: chemoradiotherapy.

In the 264 patients, serum levels of albumin and amylase, CA19-9, TS, MPDD, and PPV were significantly decreased after CRT, compared with those before CRT. In the 44 patients who had plasma levels of apoAII isoforms measured, albumin, CA19-9, TS, and PPV significantly decreased after CRT (Table 1). Focusing on the indices for the assessment of nutritional status, albumin, total lymphocyte, and PNI were significantly decreased after CRT, although total cholesterol did not change.  Figure 2 shows a case that demonstrated drastic reduction in TS, MPDD, and PPV on the CT image after CRT. This case is a 76-year-old female with initially UR-LA PDAC in whom drastic reductions were observed not only in TS (before vs. after CRT: 59.4 vs. 52.2 mm, Figure 2(a1), (b1), (a2), (b2)) but also in MPDD (7.6 vs. 3.7 mm) as well as PPV (39.8 vs. 29.3 ml) (Figure 2(a3), (b3)).

### 3.2. Study II: Alteration of Plasma ApoAII Isoforms before and after CRT

#### 3.2.1. Distribution of ApoAII-ATQ/AT and Two-Dimensional Scatter Graph of ApoAII-ATQ and -AT

**Figure 3:**
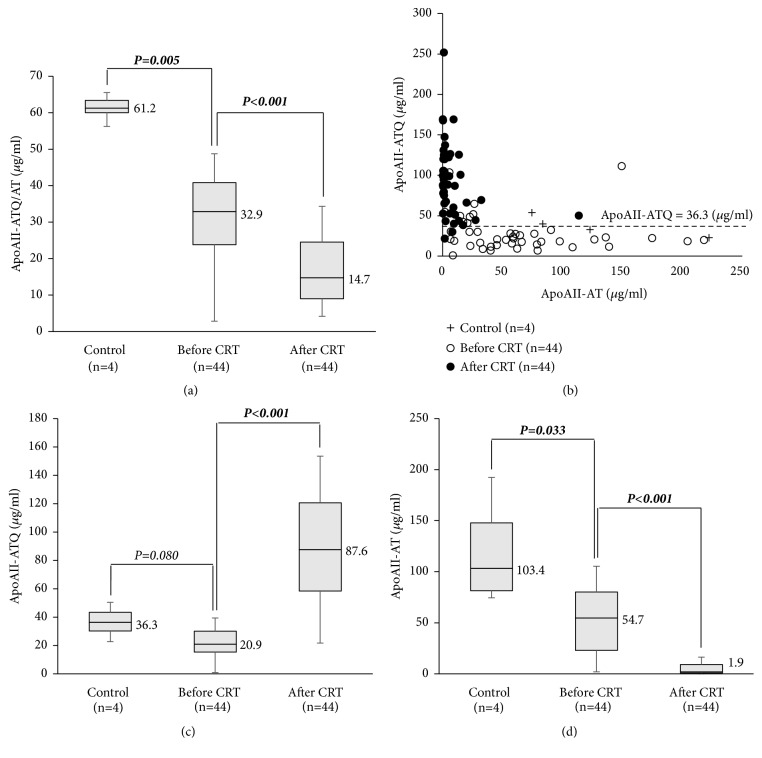
Distribution of apoAII-ATQ/AT and two-dimensional scatter graph of apoAII-ATQ and -AT in healthy controls and PDAC patients treated with CRT. (a) Distribution of apoAII-ATQ/AT in healthy controls (n=4) and the patients treated with CRT (n=44). (b) Two-dimensional scatter graphs of apoAII-ATQ and -AT in healthy controls (n=4) and pancreatic cancer patients treated with CRT (n=44). (c) Distribution of apoAII-ATQ in healthy controls (n=4) and the patients treated with CRT (n=44). (d) Distribution of apoAII-AT in healthy controls (n=4) and the patients treated with CRT (n=44). PDAC: pancreatic ductal adenocarcinoma and CRT: chemoradiotherapy.

Plasma levels of apoAII-ATQ/AT (*μ*g/ml) at the time before CRT in the 44 PDAC patients were significantly lower than those in 4 healthy controls (median, 32.9 vs. 61.2, P=0.005), and after CRT these levels were significantly decreased from 32.9 to 14.7 (*P*<0.001) (Figure 3(a)). As shown in two-dimensional scatter graph in Figure 3(b), the distribution of apoAII isoforms (apoAII-ATQ and -AT) before CRT (open circle) had dramatically changed after CRT (closed circle): open circles were scattered along the x-axis of apoAII-AT, showing apoAII-ATQ level (unprocessed apoAII-isoform) of 20.9 and apoAII-AT level of 54.7, while closed circles were scattered along the y-axis of apoAII-ATQ, showing high apoAII-ATQ level of 87.6 and very low apoAII-AT level of 1.9. The dotted line in Figure 3(b) was the median level of apoAII-ATQ in healthy controls (36.3 *μ*g/ml). Given these results, the hyper- and hypoprocessing patterns were defined as the two groups whose apoAII-ATQ levels were less than 36.3 and 36.3 or more. Among the 44 patients, 35 (79.5%) showed hyperprocessing pattern before CRT. Among 32 patients whose apoAII-ATQ/AT was decreased after CRT, 30 patients (93.8%) showed hypoprocessing pattern after CRT.

At the time before CRT, the levels of apoAII-ATQ and -AT were both significantly lower in the PDAC patients before CRT than in the healthy controls: 20.9 vs. 36.3 (*P*=0.080) and 54.7 vs. 103.4 (*P*=0.033), which reflected that the PDAC patients demonstrated the hyperprocessing pattern (Figures 3(c) and 3(d)). After CRT, apoAII-ATQ levels were significantly increased (20.9 vs. 87.6,* P*<0.001), while apoAII-AT levels were significantly decreased (54.7 vs. 1.9,* P*<0.001). These results indicated that CRT altered the direction of proteolytic digestion from the hyper- to hypoprocessing pattern in the PDAC patients.

#### 3.2.2. Relationship between Alteration of ApoAII-ATQ/AT and Clinical/Histological Effect of CRT

**Figure 4:**
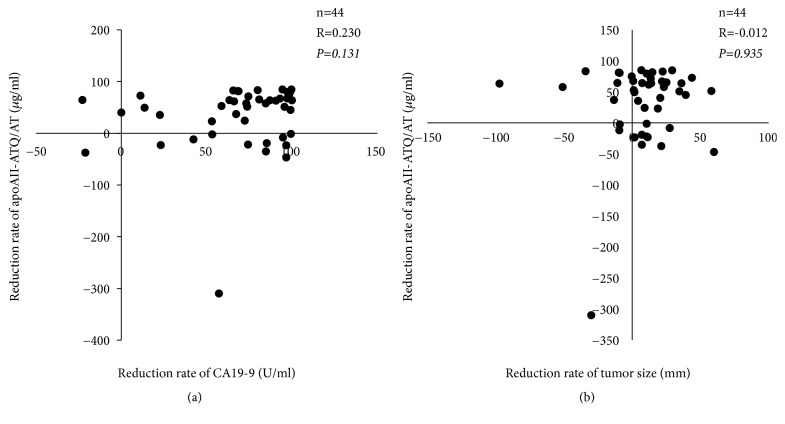
Relationship between alteration of apoAII-ATQ/AT and clinical effect of chemoradiotherapy. (a) Correlation between reduction of serum CA19-9 level and that of apoAII-ATQ/AT (n=44). (b) Correlation between reduction of tumor size and that of apoAII-ATQ/AT (n=44). Tumor size: sum of short and long axis.

As for the reduction rates of apoAII-ATQ/AT after CRT, they were not significantly correlated with either those of serum CA19-9 levels or TS (R=0.230 and -0.012,* P*=0.131 and 0.935, respectively) (Figure 4). When histological response of the CRT was examined and classified into the high responders (tumor destruction of >50%) and low responders (tumor destruction of ≤50%) [20], among 13 patients with resection, there were low responder in 5 (38.5%) and high responder in 8 (61.5%). The reduction rates of apoAII-ATQ/TQ (%) after CRT in low and high responders were 67.1% (-2.3-81.2) and 54.4% (-22.8-84.7), respectively, showing no significant difference between the two groups.

#### 3.2.3. Plasma Levels of ApoAII-ATQ/AT, -ATQ, and -AT according to MPDD and PPV after CRT

**Figure 5:**
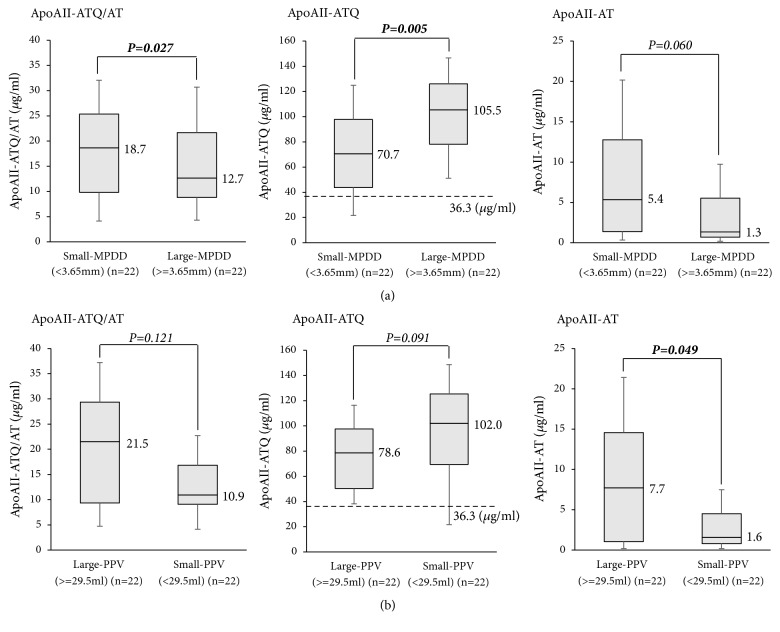
*Levels of apoAII-ATQ/AT, apoAII-ATQ, and apoAII*-*AT in the PDAC patients according to MPDD and PPV after CRT.* (a) Comparison of distribution of apoAII-ATQ/AT, apoAII-ATQ, and apoAII-AT between the patients with small MPDD and those with large MPDD after CRT. The 44 PDAC patients were divided into small- or large-MPDD groups at the median level of MPDD (3.65 mm) after CRT. (b) Comparison of distribution of apoAII-ATQ/AT, apoAII-ATQ, and apoAII-AT between the patients with large PPV and those with small PPV after CRT. The 44 PDAC patients were divided into small- or large-PPV groups at the median level of PPV (29.5 ml) after CRT. PDAC: pancreatic ductal adenocarcinoma, MPDD: main pancreatic duct diameter, PPV: pancreatic parenchymal volume excluding tumor volume, and CRT: chemoradiotherapy.

When the 44 patients were divided into large- (3.65 mm or more) and small-MPDD (less than 3.65 mm) groups at its median value after CRT (3.65 mm, these data were rounded off to the first decimal place in Table 1), the large-MPDD group showed markedly lower apoAII-ATQ/AT, higher apoAII-ATQ, and lower apoAII-AT than those of the small-MPDD group (12.7, 105.5, and 1.3 vs. 18.7, 70.7, and 5.4,* P*= 0.027, 0.005, and 0.060, respectively) (Figure 5(a)). When the patients were divided into large- (29.5 ml or more) and small-PPV (less than 29.5 ml) groups at its median value after CRT (29.5 ml), the small-PPV group showed lower apoAII-ATQ/AT, higher apoAII-ATQ, and lower apoAII-AT than the large-PPV group (10.9, 102.0, and 1.6 vs. 21.5, 78.6, and 7.7,* P*=0.121, 0.091, and 0.049, respectively) (Figure 5(b)). According to the above results, large-MPDD and small-PPV groups were in the state of more advanced hypoprocessing pattern, suggesting advanced pancreatic exocrine disorder. The large-MPDD and small-PPV groups showed lower serum levels of amylase after CRT than small-MPDD and large-PPV group, respectively, although there was no significant difference between each other, supporting that pancreatic exocrine function was decreased in large-MPDD and small-PPV group.

#### 3.2.4. Alteration of ApoAII-ATQ/AT, -ATQ, and -AT in the Plasma of PDAC Patients after CRT by Incubating with Human Pancreatic Juice

**Figure 6:**
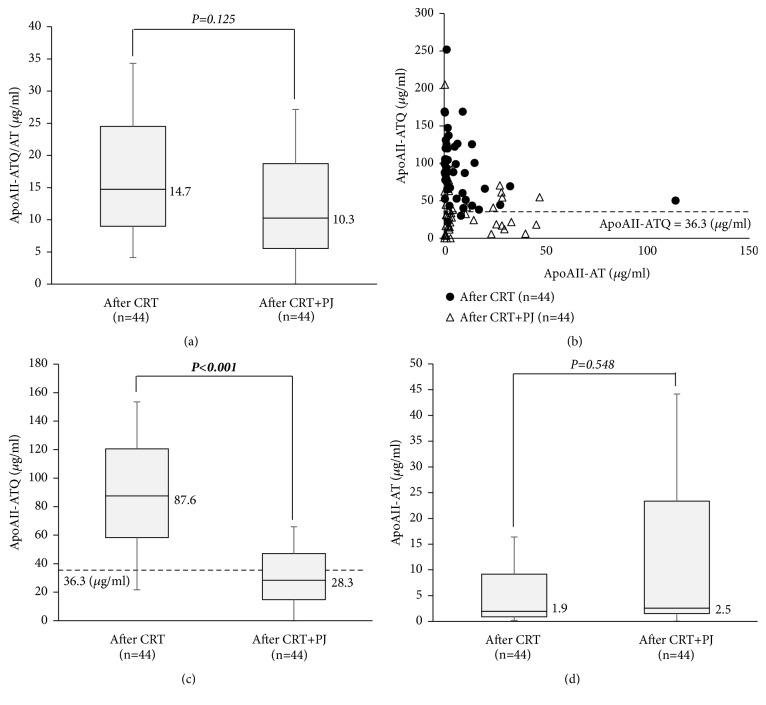
*Alteration of apoAII isoforms after the incubation with pancreatic juice in PDAC patients after CRT (n=44)*. PDAC: pancreatic ductal adenocarcinoma, CRT: chemoradiotherapy, and PJ: pancreatic juice.

To elucidate the underlying mechanism of the alteration of apoAII-ATQ/AT from the perspective of pancreatic exocrine function, plasma samples after CRT were incubated with PJ that was obtained from the one patient without PDAC. After the incubation, plasma levels of apoAII-ATQ/AT were not significantly changed (median, 14.7 vs. 10.3,* P*=0.125) (Figure 6(a)). However, the alterations of apoAII-ATQ and -AT between after CRT and after CRT+PJ were inverse from those between before and after CRT. As shown in the two-dimensional scatter graph in Figure 6(b), the distribution of apoAII isoforms (apoAII-ATQ and -AT) before incubation, that is, after CRT (closed circle), had dramatically changed after incubation, that is, after CRT + PJ (open circle). After incubation, apoAII-ATQ levels were significantly decreased (87.6 vs. 28.3,* P*<0.001), and apoAII-AT levels were not significantly changed (1.9 vs. 2.5,* P*=0.548), indicating that incubation with human pancreatic juice could alter the direction of proteolytic digestion from hypoprocessing to hyperprocessing pattern in the serum apoAII isoforms of the PDAC patients at the time after CRT (Figures 6(c) and 6(d)).

## 4. Discussion

The current study revealed that the distribution of apoAII isoforms showed drastic changes between before and after CRT for the PDAC patients, which were not correlated with treatment effect of CRT but rather with the changes in morphological features of the pancreas. As the previous studies had already demonstrated, the plasma apoAII-ATQ/AT levels [represented as the plasma apoAII√(ATQ*∗*AT)] in the current study were significantly lower in the PDAC patients than in the healthy controls, suggesting that apoAII-ATQ/AT is a promising marker for detection of PDAC [2, 3]. Therefore, it had been anticipated that plasma apoAII-ATQ/AT after CRT might be significantly increased according to the degree of treatment response to CRT and that apoAII-ATQ/AT could be used as a surrogate marker for the effect of CRT, if it is a tumor-specific biomarker. Contrary to our expectations, the levels of apoAII-ATQ/AT were significantly decreased after CRT, compared with those before CRT, although the serum CA19-9 levels and TS showed a great reduction after CRT. Additionally, the alterations in serum CA19-9 levels and the clinical efficacy of CRT assessed by TS were not correlated with those in apoAII-ATQ/AT, suggesting that this newly established biomarker is not a tumor-specific biomarker.

Pancreatic morphological features such as pancreatic atrophy and change of MPDD are considered a unique radiographic parameter that reflects pancreatic exocrine function. Several clinical studies have reported that a close correlation exists between pancreatic exocrine function and morphology of pancreas in the patients with chronic pancreatitis as well as in those who underwent pancreatectomy [12, 16, 17]. The current study clearly demonstrated that MPDD and PPV excluding tumor after CRT significantly decreased, compared with those before CRT. To the best of our knowledge, the current study is the first report clearly demonstrating that CRT for PDAC significantly reduces not only TS but also PPV as well as MPDD, although there have been a lot of studies on changes of tumor size after chemotherapy or CRT for PDAC [21–23]. A causal relationship between irradiation to the pancreas and change of pancreatic morphology has been suggested [24]. The previous abdominal radiotherapy for Hodgkin' disease or seminoma is known to be a cause for chronic pancreatitis [25]. A diffuse pancreatic atrophy similar to chronic pancreatitis was reported as one of morphological features which was caused by irradiation to the pancreas based on the imaging findings [24]. The mechanism of radiation injury to the pancreas is generally considered to be vascular damage. It was reported that intraoperative radiotherapy for pancreas caused acinar cell degranulation, connective tissue fibrosis, pancreatic duct damage, and blood vessel damage, which occurs in endothelial cells and connective tissue of media and adventitia that bring lumen occlusion [26]. It seems most likely that the degree of radiation injury to the pancreas depends on the method of radiation delivery, dose of radiation, volume of irradiated tissue, and frequency of radiation fractions. In the current study, patients underwent three-dimensional conformal radiotherapy in which the normal surrounding tissues such as extrapancreatic nerve plexus and regional lymph nodes as well as the intended tumor were included in the primary radiation target. Therefore, even if the pancreatic parenchyma excluding the intended tumor is hardly affected by the three-dimensional conformal radiotherapy, irradiation to the surrounding peripancreatic soft tissue might cause acinar cell degranulation most likely due to vascular process, which consequently causes pancreatic atrophy.

There are five circulating apoAII isoforms; apoAII-ATQ/ATQ, -ATQ/AT, -AT/AT, -AT/A, and -A/A, which are characterized by the truncation of varying numbers of amino acids from the C-terminus of the apoAII homo/heterodimer. The initial cleavage of a C-terminal glutamine (Q) in apoAII-ATQ/ATQ by carboxypeptidases most likely primes apoAII protein to further proteolytic digestion, and this process forms five circulating apoAII isoforms in the above order as shown in Figure 1. Among carboxypeptidases, carboxypeptidase A is a digestive enzyme that is primarily synthesized by the pancreas [3]. The following two processing patterns were defined based on the median plasma level of apoAII-ATQ in healthy controls: (1) hyperprocessing patterns in which the apoAII-ATQ level was very low reflecting large amount of consumption of apoAII-ATQ/ATQ and the light isoform apoAII–A/A were predominantly observed (although apoAII-A/A could not be measured) and (2) hypoprocessing patterns in which the apoAII-ATQ level was very high reflecting little consumption of apoAII-ATQ/ATQ and the heavy isoform apoAII-ATQ/ATQ were predominantly observed.

Both hyper- and hypoprocessing patterns are recognized in PDAC patients. In contrast, neither hyper- nor hypoprocessing patterns were observed in healthy controls, and hyper- and hypoprocessing patterns were never observed in other gastroenterological diseases [4], although the definition of hyper- and hypoprocessing patterns based on the median plasma apoAII-ATQ of healthy controls was original in the current study and could not be applied to previous reports. Therefore, these two processing patterns were considered to be unique phenomena to pancreatic diseases which might be explained by the activity of carboxypeptidases released from the pancreas into the blood stream, and it seems most likely that the distribution of apoAII-ATQ/AT represents the status of pancreatic exocrine secretion. Consequently, high internal pressure of main pancreatic duct due to obstruction by tumor invasion promotes release of circulating pancreatic enzymes, such as carboxypeptidase A, which reduces serum level of apoAII-ATQ/ATQ, indicating hyperprocessing pattern. The concomitant obstructive pancreatitis also gradually progresses and leads to pancreatic atrophy and impairment of pancreatic exocrine function which decelerate release of circulating pancreatic enzymes from the pancreas, which consequently reduces serum level of apoAII-AT/AT, indicating hypoprocessing pattern.

The significant changes of both pancreatic morphology and plasma apoAII-ATQ/AT after CRT led us to speculate that the rapid change in pancreatic exocrine function after CRT affected the alteration of plasma apoAII-ATQ/AT level. After CRT, apoAII-ATQ significantly increased, while apoAII-AT significantly decreased, indicating that the distribution of apoAII isoforms shifted from mixed hyper- and hypoprocessing pattern to predominant hypoprocessing pattern. The alteration of apoAII processing during CRT synchronized with a drastic atrophic change of pancreas most likely caused by CRT. It was considered that the drastic atrophic change of pancreas assessed by PPV reflected pancreatic exocrine dysfunction, causing a reduction of circulating pancreatic enzymes which in turn might cause decreased cleavage of the apoAII C-terminal glutamine (Q) and pooling of apoAII-ATQ/ATQ. According to the review by the pathologist (K.U.) in the resected specimen after CRT, we confirmed that almost all patients showed marked parenchymal fibrosis and significantly decreased number of acinar cells (data not shown), indicating that marked alteration of apoAII isoforms from hyperprocessing to hypoprocessing pattern during CRT is associated with rapid progression of pancreatic exocrine dysfunction caused by CRT.

The distribution of apoAII isoforms was associated with the PPV and MPDD, both of which were considered to reflect pancreatic exocrine function. It has been reported that a close correlation exists between pancreatic exocrine function and morphology of pancreas in the patients who had chronic pancreatitis or in those who underwent pancreatectomy [12, 16, 17]. The result of the current study, that serum amylase level was lower in large-MPDD and small-PPV than in small-MPDD and large-PPV groups, supported these previous reports. As for the association between pancreatic morphological features and apoAII isoforms after CRT, the results of the current study supported the underlying mechanism for alteration in apoAII isoforms in PDAC patients who were treated with CRT. In the patients with large MPDD and small PPV after CRT who were considered to have impaired pancreatic exocrine function, apoAII-ATQ was significantly higher, while apoAII-AT was significantly lower compared with those with small MPDD and large PPV after CRT. These results suggested that decreased circulating pancreatic enzyme released from pancreas in the patients with large MPDD and small PPV after CRT could be attributed to a lack of cleavage of plasma apoAII-ATQ/ATQ. Consequently, the PDAC patients who were treated with CRT, especially with large MPDD and small PPV, predominantly showed a hypoprocessing pattern.

To confirm this hypothesis, we further examined the alterations of apoAII isoforms by incubating the plasma samples after CRT with human PJ obtained from the one patient who underwent pancreaticoduodenectomy for branch-duct IPMN. After the incubation with PJ, the distribution of apoAII isoforms was changed from hypoprocessing pattern to hyperprocessing pattern, showing significant reduction in plasma apoAII-ATQ and a little increase in plasma apoAII-AT. Plasma apoAII-ATQ/AT was expected to increase after incubation; however, contrary to our expectations, it did not increase after incubation. The cause of this phenomenon was considered that the increase of apoAII-AT was little because further proteolytic digestion progressed and then apoAII-AT/A and -A/A were produced rather than apoAII-ATQ/AT and -AT/AT, although the plasma apoAII-A could not be measured. Taken together, addition of pancreatic enzymes to the plasma of PDAC patient after CRT restored the processing of apoAII isoforms.

The current study has several limitations. First, pancreatic exocrine function was evaluated using several serum markers such as amylase, albumin, and total cholesterol, especially amylase, which do not precisely reflect the pancreatic exocrine function. It should be evaluated more precisely using the examinations such as N-benzoyl-L-tyrosyl-p-amino-benzoic acid (BT-PABA) excretion test, 13C-labeled mixed triglyceride breath test, and magnetic resonance cholangiopancreatography with secretin stimulation [27–31]. Second, in these situations, symptoms such as diarrhea or nutritional status should be assessed as a surrogate endpoint for pancreatic exocrine function. Therefore, we examined the presence and frequency of diarrhea; however, it was impossible to obtain the precise data from patient electronic medical record in a retrospective fashion. As for the nutritional status, PNI was significantly decreased after CRT, suggesting that pancreatic exocrine dysfunction after CRT caused impaired nutritional status. Third, human pancreatic juice, which was utilized in an incubation experiment, included many other substances except for the pancreatic enzyme, which might bring the alteration of apoAII isoforms after the incubation. In this experiment, the chromatographic purity of the pancreatic juice was not tested. Further study is required by measuring the activity of carboxypeptidase A itself in PJ.

In conclusion, plasma apoAII-ATQ/AT levels are not useful for evaluation of clinical effect of CRT for PDAC, but their isoforms are very useful to assess pancreatic exocrine disorder because pancreatic atrophy and insufficient secretion of circulating pancreatic enzymes after CRT are considered likely to influence apoAII-ATQ levels. The current study contributes to comprehension of the underlying mechanism of the alteration of apoAII-ATQ/AT in PDAC patients who underwent CRT. Before CRT, most patients with PDAC were considered to be in the state of hyperprocessing pattern of apoAII isoforms which might reflect the increased circulating pancreatic enzymes caused by main pancreatic duct obstruction, while after CRT most of them were shifted to the state of hypoprocessing pattern which might reflect the decreased circulating pancreatic enzymes caused by pancreatic atrophy. Furthermore, clinical application of apoAII isoforms is anticipated not only for early detection of PDAC and pancreatic malignancy but also for monitoring pancreatic exocrine function in patients with pancreatic diseases.

## Figures and Tables

**Table 1:** Serum pancreatic functional marker, clinical effect of CRT, and pancreatic morphological features before and after CRT.

The 264 patients with PDAC who were enrolled in CRT protocol from February 2005 to May 2016			
	Before CRT	After CRT	*P value*
Albumin (g/dl) (n=260)	3.9 (2.5-4.7)	3.8 (2.2-4.7)	***0.025***
Total cholesterol (mg/dl) (n=210)	162 (36-326)	158 (74-251)	*0.114*
Amylase (U/l) (n=220)	74 (16-651)	59 (9-808)	***<0.001***
CA19-9 (U/ml)	180.2 (0.1-26666.4)	43.5 (0.1-9476)	***<0.001***
TS (mm)	58.1 (13.6-137.6)	55.6 (19.8-312.1)	***<0.001***
MPDD (mm)	4.0 (0.3-15.5)	3.6 (0.5-16.3)	***<0.001***
PPV (ml)	34.8 (3.4-97.8)	25.2 (3.6-85.2)	***<0.001***

The 44 patients with PDAC who had apoAII isoforms measured from February 2015 to May 2016			
	Before CRT	After CRT	P value

Albumin (g/dl)	3.9 (3.1-4.6)	3.7 (2.7-4.4)	***0.038***
Total lymphocyte (mm^3^)	1400 (480-3860)	805 (220-2060)	***<0.001***
Total cholesterol (mg/dl) (n=27)	155 (103-199)	167 (99-219)	*0.178*
Prognostic nutritional index	45.6 (32.9-57.4)	41.5 (31.1-52)	***<0.001***
Amylase (U/l) (n=36)	71 (28-248)	59 (18-249)	*0.346*
CA19-9 (U/ml)	229.7 (0.1-26666.4)	28.5 (0.1-2228.5)	***<0.001***
TS (mm)	60.9 (23.4-137.6)	56.5 (25.7-119.8)	***0.002***
MPDD (mm)	3.7 (1.0-12.5)	3.7 (0.7-11.5)	*0.511*
PPV (ml)	37.0 (15.3-82.9)	29.5 (8.1-69.1)	***<0.001***

CRT: chemoradiotherapy, TS: tumor size (sum of short and long axis), MPDD: diameter of main pancreatic duct, and PPV: pancreatic parenchymal volume excluding tumor volume

## Data Availability

The data used to support the findings of this study are available from the corresponding author upon reasonable request.
